# Gender Norms and Structural Barriers to Use of HIV Prevention in Unmarried and Married Young Women in Manicaland, Zimbabwe: An HIV Prevention Cascade Analysis

**DOI:** 10.12688/gatesopenres.15127.1

**Published:** 2024-03-07

**Authors:** Simon Gregson, Louisa Moorhouse, Rufurwokuda Maswera, Tawanda Dadirai, Phyllis Mandizvidza, Morten Skovdal, Constance Nyamukapa

**Affiliations:** 1 School of Public Health, Imperial College London, London, England, W2 1PG, UK; 2 Biomedical Research and Training Institute, Harare, Harare Province, Zimbabwe; 3 Department of Public Health, University of Copenhagen, Copenhagen, Capital Region of Denmark, Denmark

**Keywords:** HIV, AGYW, HIV prevention cascades, Social norms, Structural barriers, Zimbabwe

## Abstract

**Background:**

Gender norms against adolescent girls and young women (AGYW)’s having pre-marital sex and using condoms in marriage are included as barriers to motivation to use condoms in HIV prevention cascades. Representative survey data on gender norms are needed to test this assumption.

**Methods:**

General-population survey participants in Manicaland, Zimbabwe (ages≥15, N=9803) were asked if they agreed/disagreed with statements on gender norms. AGYW at risk of HIV infection were asked whether community views discouraged condom use. Multivariable logistic regression was used to measure variations in community members’ views and associations between AGYW’s perceiving negative gender norms and condom HIV prevention cascades.

**Results:**

57% of men and 70% of women disagreed that ‘If I have a teenage daughter and she has sex before marriage, I would be ok with this’; and 41% of men and 57% of women disagreed that ‘If I have a teenage daughter, I would tell her about condoms’. 32% and 69% of sexually-active HIV-negative unmarried AGYW, respectively, said negative community views were important in decisions to use condoms and their friends were not using condoms. In each case, those who agreed had lower motivation to use condoms. Fewer of those with friends not using condoms reported using condoms themselves (39% *vs.* 68%; p<0.001). 21% of men and 32.5% of women found condom use in marriage acceptable. 74% and 93% of married AGYW at risk, respectively, said negative community views influenced their decisions to use condoms and their friends did not use condoms. Fewer married AGYW reporting friends not using condoms were motivated to use condoms but there was no difference in reported condom use (4.1% *vs.* 6.9%; p=0.48).

**Conclusions:**

Negative gender norms can form a barrier to motivation to use condoms in unmarried and married AGYW at risk of HIV infection, and, for unmarried AGYW, to condom use.

## Introduction

Social norms can be an important structural determinant of demographic behaviour ^1^. Furthermore, they often evolve over time in nature and influence ^2^ and may be amenable to intervention ^3^. Therefore, it is unfortunate that social norms are measured only infrequently in population surveys. In HIV control in sub-Saharan Africa, gender-based social norms are thought to be a key driver of patterns of sexual activity that place adolescent girls and young women (aged 15–24; AGYW) at high risk of infection ^4^. However, few – if any – studies have collected and used representative population survey data to characterise and help understand the local dynamics of support for and compliance with these gender norms and their links to HIV infection and associated unprotected sexual risk behaviours. 

Rates of new HIV infections have fallen in many sub-Saharan African populations in recent years ^5^. However, these declines have resulted more from reductions in viral load and infectiousness in people living with HIV, due to high antiretroviral treatment coverage ^6^, than from reductions in unprotected sexual risk behaviours amongst uninfected individuals ^7^. HIV incidence in AGYW is still high and infection levels remain considerably higher in young women than in young men ^8^. A number of individual-level socio-demographic, behavioural and biological risk factors have been associated with HIV risk in AGYW. These include early school drop-out, low education and alcohol use ^9^; early age at first sex, early marriage, age-disparate sexual relationships and multiple sexual partners ^10, 11^; having an uncircumcised partner and inconsistent condom use ^12^; and infection with – or having a partner with – another sexually transmitted disease ^13^. Whilst interventions aimed at addressing individual-level barriers to HIV prevention have achieved statistically significant reductions in young people’s HIV risk behaviours ^14^, the extent and longevity of these reductions have not been sufficient to reduce rates of new HIV infections ^15^.

Increasingly, attention has turned to understanding ^16, 17^ and, where necessary, intervening on ^3, 7, 14, 18^ the structural factors that contextualise the individual-level behaviours that place AGYW at higher risk of HIV infection. These structural factors include the gender identities and the gendered social norms that surround early sexual activity (Jewkes, JIAS, 2010). This work (quite rightly) has concentrated largely on removing harmful male gender norms and on bringing about gender power equity (Pulerwitz, 2008; Pulerwitz, 2019; Pettifor, 2018; Jewkes, 2010) but it may be useful also to consider the nature and effects of inter-related social norms that surround early female sexual activity.

It is believed that gender norms on young women’s early sexual activity in sub-Saharan African countries with large-scale HIV epidemics pose particular barriers to their use of condoms for HIV prevention both before and during marriage. However, the reality that many AGYW are already in marital unions is not always considered in HIV prevention programmes which is important since the norms that affect sexual activity and use of condoms may differ greatly for unmarried and married young women. Before marriage, there has been a widespread norm against young women’s starting sex and a belief that providing information about condoms or making them available in schools will encourage this practice ^19– 21^. Despite this traditional norm, female sex before marriage has become widespread in many sub-Saharan African populations ^22^ due to pressure from boyfriends and peer pressure to accept the potential material and financial benefits that can make it possible to be perceived as modern and fashionable ^23^. As a consequence of this, unmarried AGYW now are often at risk of HIV infection through their engaging in sexual relationships with older or multiple partners; although, in some cases, this risk can be mitigated by their partners’ willingness to use condoms to prevent pre-marital pregnancies ^10^. Qualitative work in Western Kenya also suggests that, in some communities in sub-Saharan Africa, attitudes towards unmarried AGYW’s access to condoms have become divided and the social norm against making condoms available could be weakening ^20^. In contrast, once married, AGYW are expected to be sexually active and not using condoms both to satisfy their husbands and to prove their fertility to their husbands’ families ^24^. Although much of married AGYW’s risk of acquiring HIV infection can come from their spouses’ extra-marital relationships rather than from their own risk behaviour, attempts by these women to initiate condom use within marriage generally would be taken as a sign of infidelity or to be inferring the husband’s infidelity ^25^, either of which would risk arguments and possible intimate partner violence ^26^. Whilst the nature and dynamics of social norms on unmarried and married AGYW’s sexual activity have been documented in qualitative studies and could differ between men and women, as yet, the extent of support and patterns of variation in support for these norms within communities have not been quantified and compared in a sub-Saharan African setting. Population survey data therefore could be helpful to shed light on the breakdown of HIV risk in AGYW between unmarried and married young women; and on the current extent and patterns of support for traditional social norms on young women’s sexual behaviour and their effects on AGYW’s use of male condoms outside and within marriage.

In this study, we elaborate the contributions of negative social norms surrounding unmarried and married AGYW’s early sexual activity and use of condoms for HIV prevention to continuing high HIV risk in this demographic group in Manicaland province, Zimbabwe – a southern African country subject to a continuing high burden of HIV infection ^27^. We focus on male condoms as currently they remain the most widely used method of primary HIV prevention in sub-Saharan African populations. We begin by comparing HIV incidence rates and the proportions of HIV-negative individuals with hypothesised higher sexual risk behaviours for infection in AGYW by marital status (unmarried (never and formerly married) *versus* currently married) and find high levels of HIV risk in both groups. We measure and examine HIV prevention cascades (HPCs) for male condoms which show that much larger gaps in motivation and capacity to use condoms effectively contributed most to the lower levels of condom use found in married women. Together with low risk perception for HIV infection ^28^, perceived lack of social acceptability is the most common factor contributing to low motivation in both groups. We describe the levels of and patterns of variation in support for social norms that could influence condom use in unmarried and married AGYW at HIV risk (AGYW ^@RISK^) in Manicaland. Support for norms against women having sex before marriage, teenage daughters having access to condoms, and condom use within marriage is mixed and varies by socio-demographic characteristics, relationship to an AGYW, community role and participation, and HIV status. We also measured unmarried and married AGYW ^@RISK^’s own perspectives on the influence of gender norms and selected other structural barriers to condom use and tested for associations with gaps in the main bars in their respective condom HPCs (i.e. lack of motivation, access or capacity to use condoms effectively). We find that, for unmarried AGYW ^@RISK^, condom use is lower in those who report that their friends do not use condoms and motivation to use condoms is lower in those who perceive community disapproval. Unmarried AGYW ^@RISK^ are also less likely to report using condoms if they experience intimate partner violence. No differences in condom use are found for married AGYW ^@RISK^ between those who report negative social norms and other structural barriers compared to those who do not.

## Methods

### Ethics

Written informed consent was obtained from all participants. For participants aged under 18, written informed consent was obtained from a parent or guardian and assent was obtained from the child. The study was approved by the Medical Research Council of Zimbabwe on 27 ^th^ April 2018 (MRCZ/A/2243) and by the Imperial College Research Ethics Committee on 20 ^th^ June 2018 (17IC4160). 

### Study setting and survey procedures

The data used in our study to measure social norms and structural factors and their influence on AGYW’s use of condoms for HIV prevention were collected in eight communities close to the Mozambique border in Manicaland, Zimbabwe’s eastern province, in a phased (one community at a time) general population survey conducted between mid-July 2018 and early December 2019. The survey started in the run-up to the first national elections to be held in the ‘New Dispensation’ period that followed the ousting of President Mugabe in November 2017 ^29^. The eight communities comprised two ‘high-density’ urban areas in Mutare, Manicaland’s provincial capital, two small towns in Nyanga and Makoni districts, two large tea and forestry plantations, and two areas of predominantly subsistence farming villages in Mutasa District. The survey provided baseline data for two community-randomised control trials of behavioural economics interventions to increase use of HIV prevention methods in AGYW (ClinicalTrials.gov registration number NCT03565575; registration date: June 20, 2018) and in young men aged 15–29 (ClinicalTrials.gov registration number NCT03565588; registration date: June 21, 2018).

The detailed survey procedures have been published previously ^30^. In brief, a census of all households in each community was carried out in one community at a time between July 2018 and November 2019. In each community, all women aged 15–24 and men aged 15–29 and random samples of two-thirds of women aged over 25 and men aged over 30 who had stayed in these households on the night before the census visit were invited to participate in detailed individual interviews. In the individual survey, participants were interviewed on a number of topics including their socio-demographic characteristics; views on AGYW’s sexual behaviour and use of HIV prevention methods; and HIV prevention cascade variables ^31^, and were tested for HIV infection. The survey questionnaire is available here.

**Figure 1.**
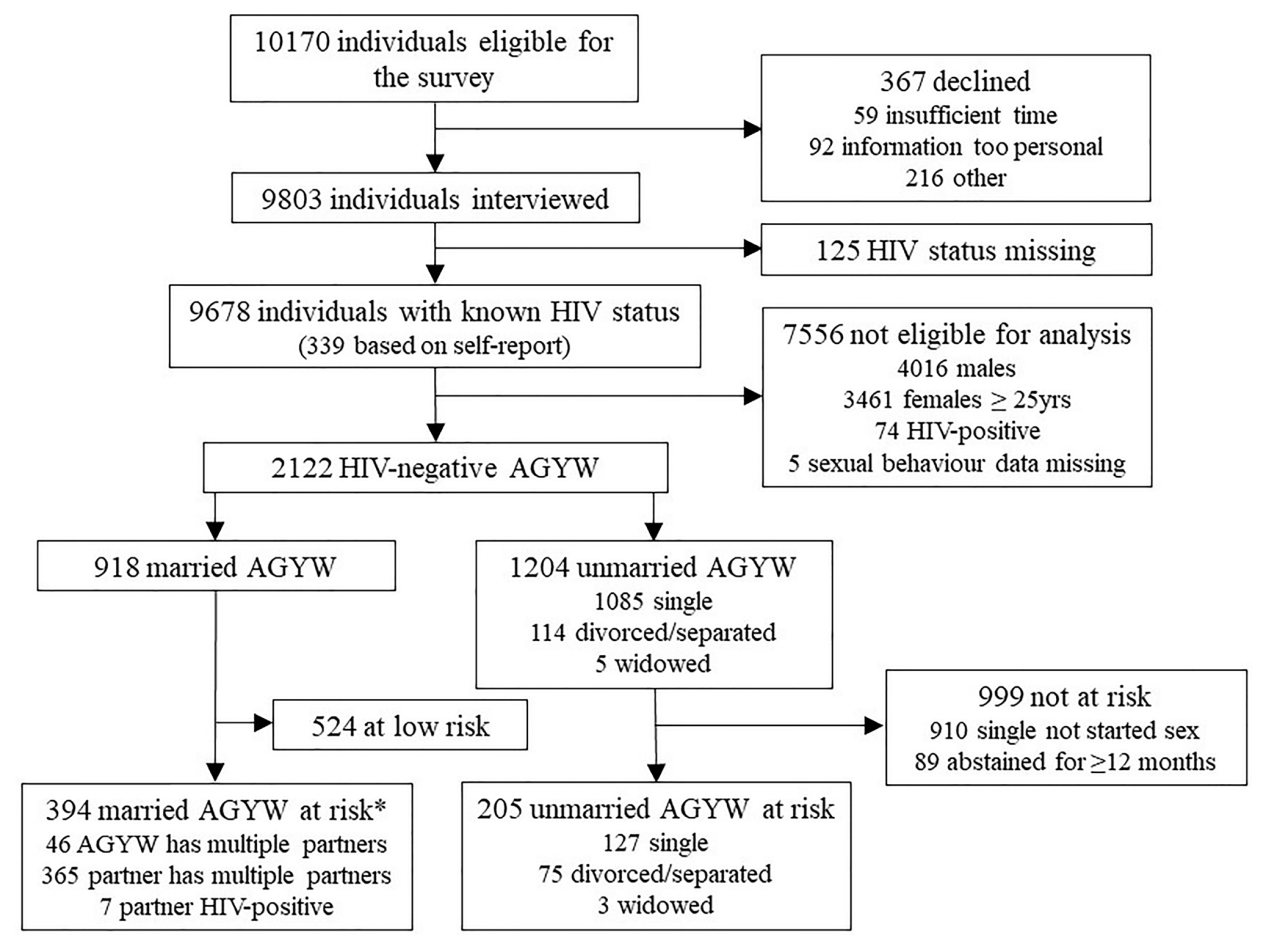
Numbers of survey participants providing data on social norms on AGYW’s sexual activity and use of HIV prevention methods and numbers of currently uninfected unmarried and married AGYW at risk of HIV infection. *19 married AGYW have other partners and a regular partner who has other partners; 5 have a partner who is HIV-positive and also has other partners.

Following these procedures, 96.4% (9803/10170) of the eligible women and men selected for the survey agreed to participate; for which, 98.7% (9678/9803) had a known HIV status either through provider-initiated testing and counselling, laboratory testing on a dried blood spot collected in the survey or, for 339 participants, by self-report ( Figure 1). Forty-two percent of survey participants were male and 58% were female. The overall weighted prevalence of HIV was 11.3% (95% CI; 10.6-12.0). HIV prevalence was higher in females (12.3%; 95% CI, 11.4-13.2) than in males (9.81%; 95% CI, 8.85-10.9).

### Analytic approach

To obtain measurements of actual risk of HIV acquisition in AGYW in Manicaland and to establish whether this risk differed by marital status and associated sexual risk behaviours, we used data from an earlier general population cohort survey (Gregson *et al.*, 2017) that ran between 1998 and 2013 in eight communities in Manicaland – six of which were also included in the 2018/19 survey. Unmarried AGYW were considered to be potentially at higher risk of acquiring HIV infection if they reported having started sex and being sexually-active in the last 12 months. Married AGYW were considered to be at potentially higher risk if they reported multiple sexual partners or sex with a spouse who was already infected or who they believed to have multiple sexual partners. HIV incidence rates in AGYW were calculated by randomly allocating seroconversion dates within the inter-survey periods that individuals became infected using multiple imputation. When calculating incidence trends over time, incidence rates were directly age-standardised to allow for changes in the age-structure of the population in the study areas over the period of the cohort, using the age-structure of Manicaland province from the 2012 Zimbabwean Population Census ^32^.

To quantify the pathways through which gender norms and other selected structural factors impact on AGYW’s use of condoms in our study population, we used data from the 2018/19 survey and the HIV prevention cascade framework proposed by Schaefer and colleagues ^31^. This framework builds from a core cascade comprising four main bars: 1) a ‘priority’ population at risk of HIV infection – and therefore in need of HIV prevention methods; 2) motivation to use an HIV prevention method amongst individuals in this priority population; 3) access to the method; and 4) effective use of the method. Each main bar in the cascade is measured conditional on inclusion in the previous bar. All survey respondents who reported effective use of the method were assumed to have been motivated and to have had access to it when they started to use it. It is considered that a gap between the last two bars in the core cascade would reflect limitations in capacity to use the method amongst individuals in the priority population who are motivated to use the method and are able to access it. The extended version of this HIV prevention cascade adds sub-bars for the gaps between each pair of consecutive main bars which represent the reasons or ‘explanatory factors’ for these gaps drawn from the theoretical and empirical literature ^33^. Lack of social acceptability to use the prevention method (e.g. due to conflicting gender norms) is included explicitly in the extended cascade as an explanatory factor that can contribute to gaps in motivation to use an HIV prevention method. In addition, negative gender norms could limit access to a method (e.g. through lack of acceptable provision if service providers disapprove of AGYW having access to HIV prevention methods ^34^) and also to capacity to use the method (e.g. by reinforcing possible partner objections to using the method). The core and extended versions of this HIV prevention cascade were measured using data collected specifically for this purpose in the population survey conducted in Manicaland in 2018/19. For further details of how the HIV prevention cascade variables were measured in this study, see *Extended data* (Tables S1–S4). Cascades were measured and compared for unmarried and married HIV-negative AGYW at higher risk of acquiring infection.

To help understand the possible influence of negative gender norms around AGYW’s sexual activity and use of HIV prevention methods, levels of support for AGYW’s pre-marital sex and access to condoms within the Manicaland population were measured in the 2018/19 survey (Q627) with results disaggregated by sex of survey respondent.

All survey participants were asked to say whether they agreed or disagreed with a series of statements ( Table 1) including the following: 

**Table 1.** Deviation from traditional gender norms on AGYW's sexual activity and use of HIV prevention methods.

Statement	Men	Women
	Proportion (95% CI)	Proportion (95% CI)
**Many young women have sex before marriage these days**	0.932 (0.924-0.939)	0.937 (0.931-0.943)
**If I have a teenage daughter and she has sex before** ** marriage, I would be OK with this**	0.428 (0.413-0.444)	0.300 (0.288-0.312)
**If I have a teenage daughter and told her not to have sex** ** until she gets married, she would comply**	0.755 (0.742-0.769)	0.656 (0.644-0.669)
**It is a good idea to make condoms available for young** ** people in schools**	0.528 (0.512-0.543)	0.488 (0.475-0.501)
**If I have a teenage daughter, I would tell her about condoms**	0.589 (0.574-0.605)	0.427 (0.414-0.440)
**If I have a teenage daughter and thought she might have ** **sex, I would encourage her to use condoms**	0.680 (0.665-0.695)	0.509 (0.496-0.523)
**If I have a teenage daughter, I would tell her about PrEP**	0.590 (0.575-0.605)	0.411 (0.399-0.424)
N	4074	5729

**Legend:** Results adjusted for under-sampling of older men (30+ years) and women (25+ years) in the survey. 95% CI: 95% confidence interval.

*“If I have / had a teenage daughter and she had sex before marriage, I would be okay with this”*
*“If I have a teenage daughter, I would tell her about condoms”*

and

*“In what circumstances is it acceptable for a husband and wife to use condoms: Always?*
*If one of them is HIV-positive?*
*If one spouse has other partners?*
*If one spouse has an STD?*
*To avoid pregnancy?”*

In the current analysis, survey participants were considered to support condom use in marriage if they agreed that this was always acceptable.

The initial results showed widespread acknowledgement that many AGYW were starting sex before marriage these days and a substantial minority of participants said they would accept it if a teenage daughter had sex before marriage. Therefore we investigated patterns of divergence from these gender norms within the community by constructing multivariable logistic regression models disaggregated by sex to calculate fully-adjusted odds ratios for agreement with the above statements by a range of socio-demographic characteristics, including the participant’s own relationship to an AGYW, community roles and HIV infection status.

Finally, to explore the pathways through which negative gender norms and selected other structural factors influence condom use by AGYW in our study population, we used multivariable logistic regression models, disaggregated by sex and adjusted for single year of age and community location, to test for statistical associations between self-reports of perceived and/or experienced negative norms and other structural factors and each of the main bars in the core HIV prevention cascade for condoms. The other structural factors included in this analysis were HIV stigma and intimate partner violence – two factors that were measured in the 2018/19 survey and have been found or suggested in the literature to limit young women’s use of HIV prevention methods ^35, 36^.

Sex differences were considered in the design of the study with sex of participants being defined based on self-report. All data analyses were done in STATA 17 (Stata Statistical Software: Release 17. College Station, TX: StataCorp LLC). R is an open-access alternative with equivalent function to STATA 17.

## Results

### HIV risk by marital status in AGYW

**Figure 2.**
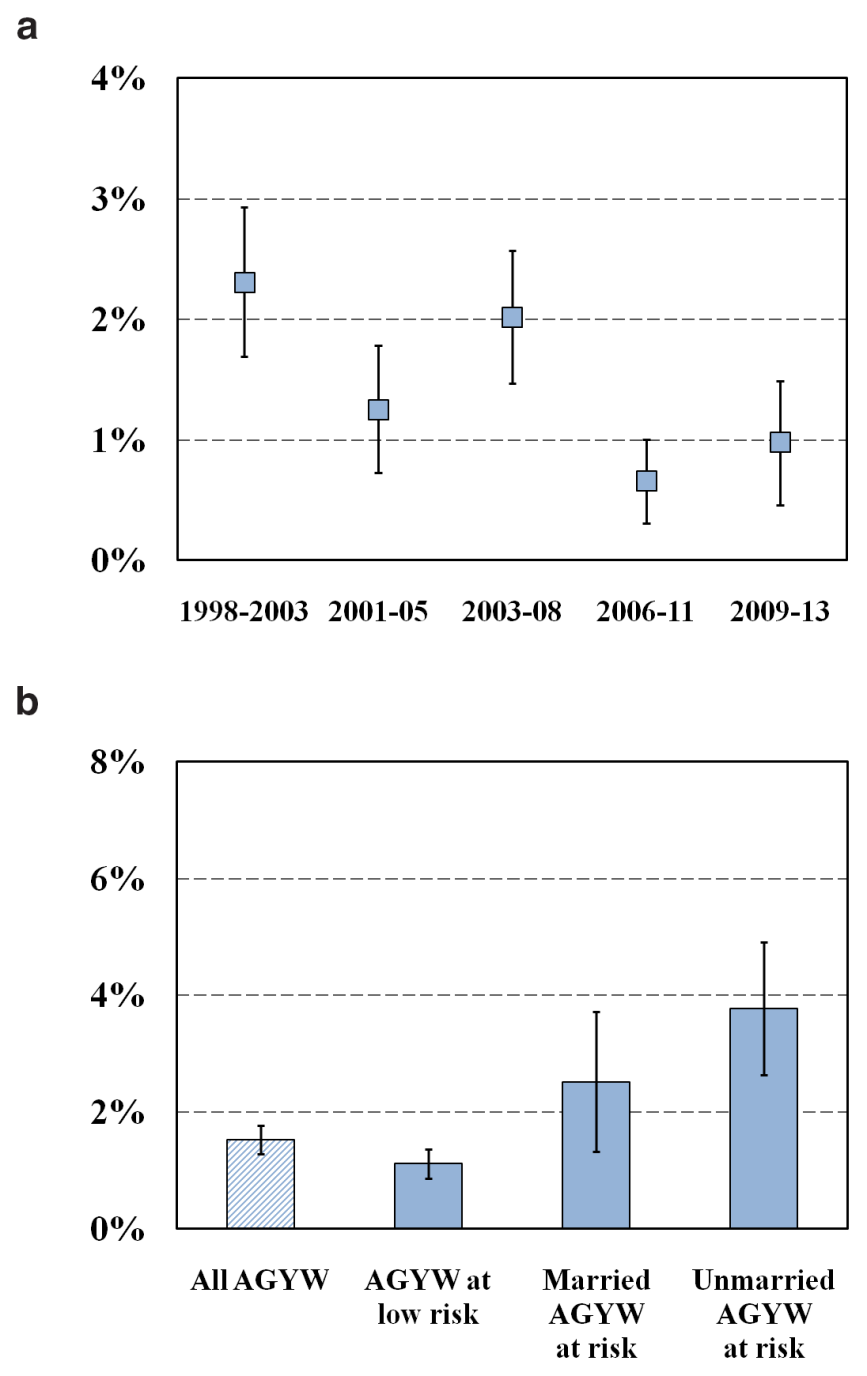
HIV incidence in AGYW, Manicaland, Zimbabwe, 1998–2013. Graph ( **a**): Trends over time in HIV incidence in AGYW. Graph ( **b**): HIV incidence in AGYW by sexual risk behaviour and marital status.

HIV incidence in AGYW in our general population cohort in east Zimbabwe declined from 2.31% (95% CI, 1.69%–2.93%) between 1998 and 2003 to 0.98% (95% CI, 0.46%–1.49%) between 2009 and 2013, with a temporary increase to 2.02% (95% CI, 1.47%–2.57%) between 2003 and 2008 ( Figure 2A) when the country experienced hyperinflation and a rapid devaluation in its national currency ^37^. Over the period of the cohort, HIV incidence was higher in sexually-active unmarried AGYW (3.77%; 95% CI, 2.63%–4.91%) and in married AGYW reporting sexual risk behaviours (2.51%; 95% CI, 1.32%–3.71%) than in other AGYW (1.11%; 95% CI, 0.85%–1.36%) ( Figure 2B).

A total of 2122 HIV-negative AGYW participated in the 2018/2019 survey ( Figure 1). Of these AGYW, 28.2% (599/2122) were at higher risk of HIV infection and therefore in the priority population for HIV prevention; 34.2% (205/599) of those at higher risk were unmarried and 65.8% (394/599) were married. Of the married AGYW at higher risk, 365 (92.6%) had a husband who had other sexual partners, 46 (11.7%) had other sexual partners themselves, and 7 (1.8%) had a husband who was HIV-positive.

### Condom HIV prevention cascades for AGYW at risk of HIV infection

**Figure 3.**
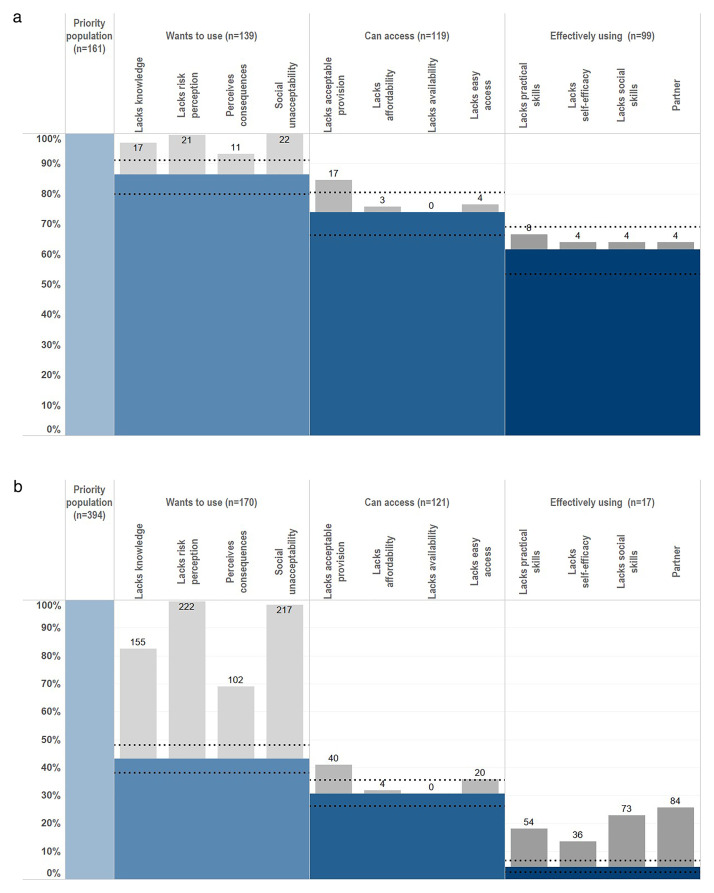
Condom HIV prevention cascades for unmarried and married AGYW at high risk of HIV infection, Manicaland, Zimbabwe, 2018/2019. Horizontal dotted lines indicate 95% confidence intervals. Graph ( **a**): Unmarried AGYW at risk. Graph ( **b**): Married AGYW at risk.

Sixty-one point five percent (95% CI, 53.5%–69.0%; N=161) of unmarried AGYW at risk and 4.3% (95% CI, 2.5%–6.8%; N=394) of married AGYW at risk used condoms at last sex. Twenty-seven point three percent (95% CI, 18.8%–37.1%; N=99) and 35.3% (95% CI, 14.2%–61.7%; N=17) of these unmarried and married AGYW who reported using condoms at last sex, respectively, also reported using condoms as a family planning method. The HIV prevention cascades for condoms show much larger gaps for married AGYW than for unmarried AGYW in motivation and in capacity to use condoms effectively (in women who were motivated and had easy access to condoms) ( Figure 3).

Eighty-six point three percent (95% CI, 80.1%–81.2%) of unmarried AGYW at risk and 43.0% (95% CI, 38.2%–48.2%) of married AGYW at risk were motivated to use condoms with their sexual partners. Amongst the 22 unmarried AGYW at risk who were not motivated to use condoms, lack of risk perception (95.5%), lack of social acceptability (100.0%), and poor knowledge about condoms (77.3%) were barriers to motivation ( Figure 3A). Amongst the 224 unmotivated married AGYW at risk, lack of risk perception (99.1%), lack of social acceptability (96.9%), and poor knowledge about condoms (69.2%) once again were the most common barriers to motivation ( Figure 3B).

Seventy-three point nine percent (95% CI, 66.4%–80.5%) of unmarried AGYW at risk and 30.7% (95% CI, 26.2%–35.5%) of married AGYW at risk were motivated and felt able to access to condoms. For both unmarried AGYW and married AGYW at risk of HIV who were motivated to use condoms, reservations about the acceptability of service provision – privacy/confidentiality concerns and being embarrassed to ask – were the most common barriers to access (unmarried AGYW: 85.0%; 17/20; married AGYW: 81.6%; 40/49). Very few of these women reported practical difficulties – long distances or limited opening hours – or high costs as barriers to accessing condoms.

Amongst the small number of unmarried AGYW at risk of HIV who were motivated to use condoms and had access to them but did not use them at last sex (N=20), limitations in partner support, self-efficacy and/or social skills all contributed to lack of capacity to use condoms ( Figure 3a). In two-fifths of these cases, lack of practical skills – not having received instruction on condom use and/or not knowing what to do when condoms break – contributed to non-use. Amongst the married AGYW at risk of HIV who were motivated to use condoms and had access to them but did not use them at last sex (N=104), lack of partner support (80.8%) and not having the social skills to discuss condom use or to refuse sex with sexual partners (70.2%) were the main constraints on capacity to use condoms ( Figure 3b). For just over a half (51.9%) of these women, lack of practical skills contributed to non-use of condoms.

### Social norms on AGYW’s sexuality and use of condoms for HIV prevention

***Social norms for unmarried AGYW.*** Almost everyone in our study population acknowledged that many young women now have sex before marriage ( Table 1). Nearly half of men (43%) and a third of women (30%) said that, if they had a teenage daughter who had sex before marriage, they would find this acceptable. Community members were split almost half-half on telling a teenage daughter – if they had or were to have one – about condoms and also pre-exposure prophylaxis (PrEP). Men were more likely than women to support this and also to support making condoms available in schools.

**Figure 4.**
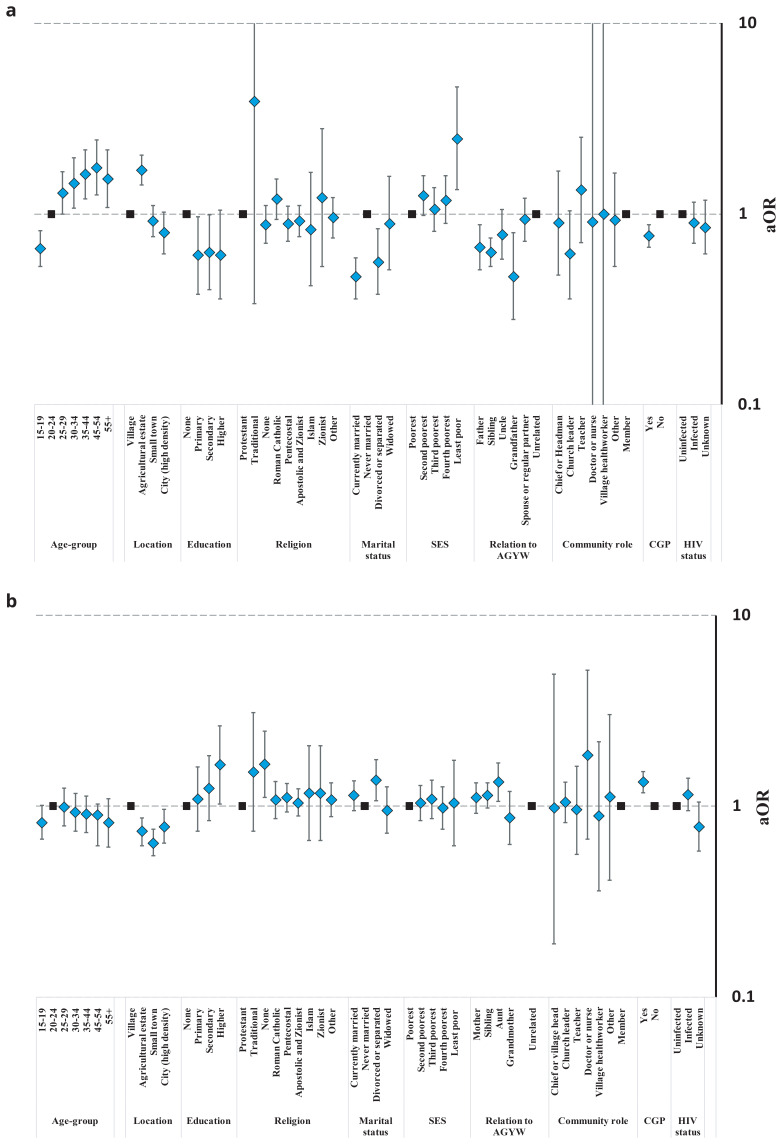
Fully-adjusted odds ratio (aOR; diamonds) and 95% confidence interval (whiskers) for acceptance of a teenage daughter having sex before marriage by community location, relationship to an AGYW, and socio-demographic characteristics, Manicaland, Zimbabwe, 2018/2019. Graph ( **a**): Male community members. Graph ( **b**): Female community members.

 Opinions on teenage daughters’ having pre-marital sex varied substantially between and within the study communities ( Figure 4, Table S5). Surprisingly perhaps, for both sexes, tolerant views towards pre-marital sex were less common in ‘high-density’ city communities (31.1%) and town communities (29.9%) than in rural subsistence farming communities (37.0%). However, men in agricultural communities were the most likely group to accept teenage daughters’ having pre-marital sex (52.2%); possibly because married men who work on these estates often live apart from their wives. Patterns of statistical association between individuals’ social roles and socio-demographic characteristics and acceptance of teenage daughters’ having pre-marital sex often differed between male and female community members. For example, approval levels increased with age for men ( Figure 4A) but decreased with age for women ( Figure 4B); never-married men were more likely to approve than currently- and formerly-married men whilst the reverse was true for women; and participation in local community groups was associated with reduced approval for men but increased approval for women. Male church leaders tended to be less likely to accept teenage daughters’ having pre-marital sex but sample sizes were too small to tell whether views varied amongst men and women performing other community roles. Men who had a teenage daughter themselves (36.9%) or who were closely related to a teenage girl at the time of the survey were less likely than other men (47.4%) to accept their daughters having sex before marriage; whilst mothers (31.7%) and aunts (36.7%) – but not grandmothers (23.2%) – tended to be more tolerant than other women (28.7%).

**Figure 5.**
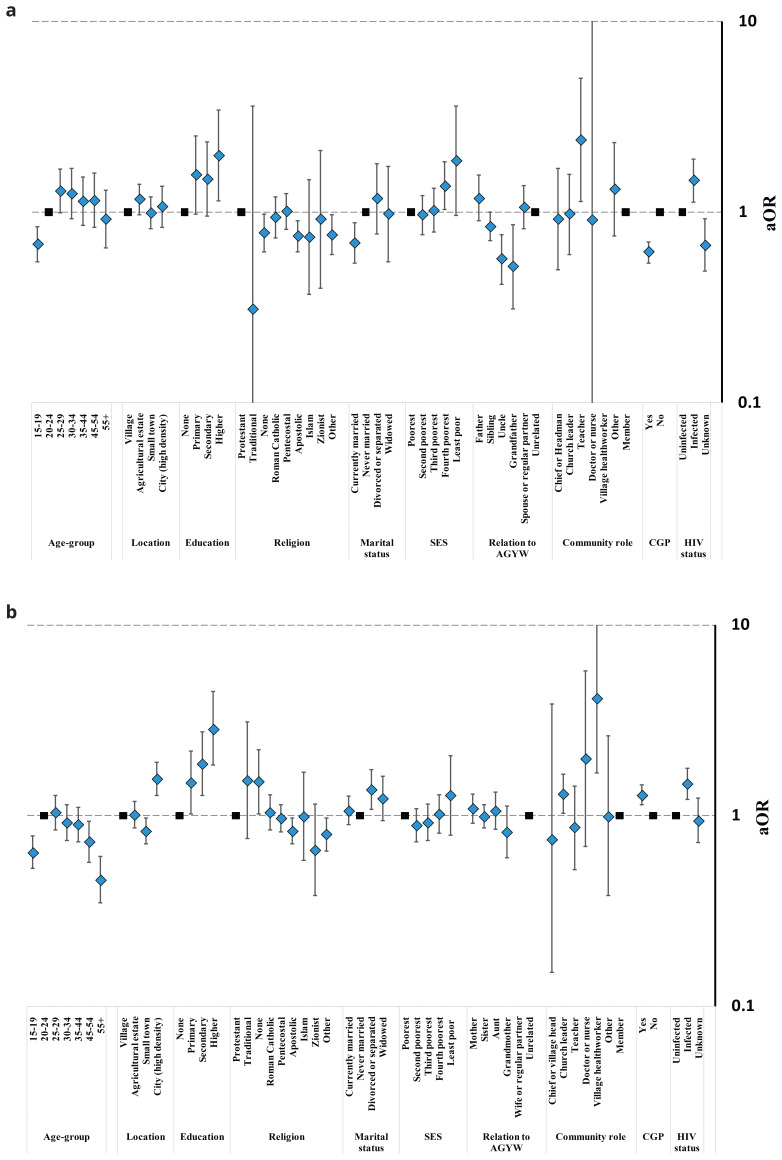
Fully-adjusted odds ratio (aOR; diamonds) and 95% confidence interval (whiskers) for approval of telling a teenage daughter about condoms by community location, relationship to an AGYW, and socio-demographic characteristics, Manicaland, Zimbabwe, 2018/2019. Graph ( **a**): Male community members. Graph ( **b**): Female community members.

 Community members views on telling teenage daughters about condoms also varied between and within the study communities ( Figure 5, Table S6). Comparing the different types of study community, men in agricultural estates (60.5%) tended to be more likely to agree that this was a good idea than those in rural communities (56.3%) (p=.10) ^1^ ( Figure 5A); possibly reflecting their more tolerant views on pre-marital sex. However, there was no evidence for this link amongst women ( Figure 5B): fewer women living in town communities (39.1%) supported telling teenage daughters about condoms than was the case in rural communities (40.1%) (p=.018) but more women in city communities (54.5%) than in rural communities supported the idea (p<.001) despite their lower tolerance of pre-marital sex. Teenagers and older people (ages 55 years and above) of both sexes were the least likely community members to support teenage daughters being told about condoms. For both men and women, those living with HIV were more likely to approve than their uninfected counterparts. Men’s agreement with telling teenage daughters about condoms varied with marital status (lowest agreement in currently married men), socio-economic status (progressively lower agreement with increasing poverty), religion (lowest agreement in Apostolic churches), and community engagement (lower agreement among men in community groups). Male teachers (78.0%) had twice the odds of agreement with telling teenage daughters about condoms as men with no particular community leadership role (58.7%) but had similar views about making condoms available in schools (Figure S1, Table S7). Women’s agreement with telling teenage daughters about condoms varied with marital status (highest agreement in divorced women), education (rising steadily with increasing education), religion (lowest agreement in Apostolic churches), and community engagement (higher agreement among women in community groups). Amongst community leaders, female village health workers had four times greater odds of agreement with telling teenage daughters about condoms compared to regular community members (72.0% *versus* 41.9%); and church leaders were also more likely to be supportive. Men who currently had a teenage daughter themselves had similar views to other men (62.8% *versus* 61.1%) on discussing condoms with daughters but brothers (55.3%), uncles (48.0%) and grandfathers (37.0%) were all less in favour ( Figure 5a). Mothers (45.3%), sisters (43.1%) and aunts (47.5%) all had similar views on this question to other women in the community (43.0%) ( Figure 5b). Grandmothers of teenage girls (25.2%) were less likely to support telling teenage girls about condoms than other women in the community (43.0%) but this difference was not statistically significant in the multivariable model (p=.21).

***Social norms for married AGYW.*** Twenty-one point three percent of men and 32.5% of women expressed the view that condoms were acceptable with a marital partner under all circumstances ( Table 2). Before accounting for other characteristics, married and unmarried men and women were equally likely to agree with this. For both sexes, condom use was most acceptable when one spouse was HIV-positive (men: 92.9%; women: 95.8%), followed by when a spouse had another sexually transmitted infection (90.2%; 94.9%), when a spouse had other sexual partners (84.4%; 91.2%), and when condoms were being used to prevent unintended pregnancies (83.0%; 79.1%). 

**Table 2.** Proportions of community members reporting agreement with circumstances when it is acceptable to use male condoms in a marital relationship.

Statement	Men		Women	
	All men	Married men	All women	Married women
	Proportion (95% CI)	Proportion (95% CI)	Proportion (95% CI)	Proportion (95% CI)
**Always**	0.213 (0.200–0.226)	0.204 (0.187–0.221)	0.325 (0.312–0.337)	0.341 (0.325–0.358)
**If one partner is HIV-positive**	0.929 (0.921–0.937)	0.942 (0.932–0.952)	0.958 (0.953–0.963)	0.969 (0.963–0.975)
**If one partner has other partners**	0.844 (0.833–0.856)	0.849 (0.834–0.865)	0.912 (0.904–0.919)	0.924 (0.915–0.933)
**If one partner has a sexually ** **transmitted infection**	0.902 (0.893–0.911)	0.913 (0.901–0.925)	0.949 (0.943–0.954)	0.963 (0.956–0.969)
**To prevent pregnancy**	0.830 (0.818–0.842)	0.814 (0.797–0.831)	0.791 (0.780–0.802)	0.794 (0.780–0.808)
N	4074		5729	

**Legend:** Results adjusted for under-sampling of older men (30+ years) and women (25+ years) in the survey. 95% CI: 95% confidence interval.

**Figure 6.**
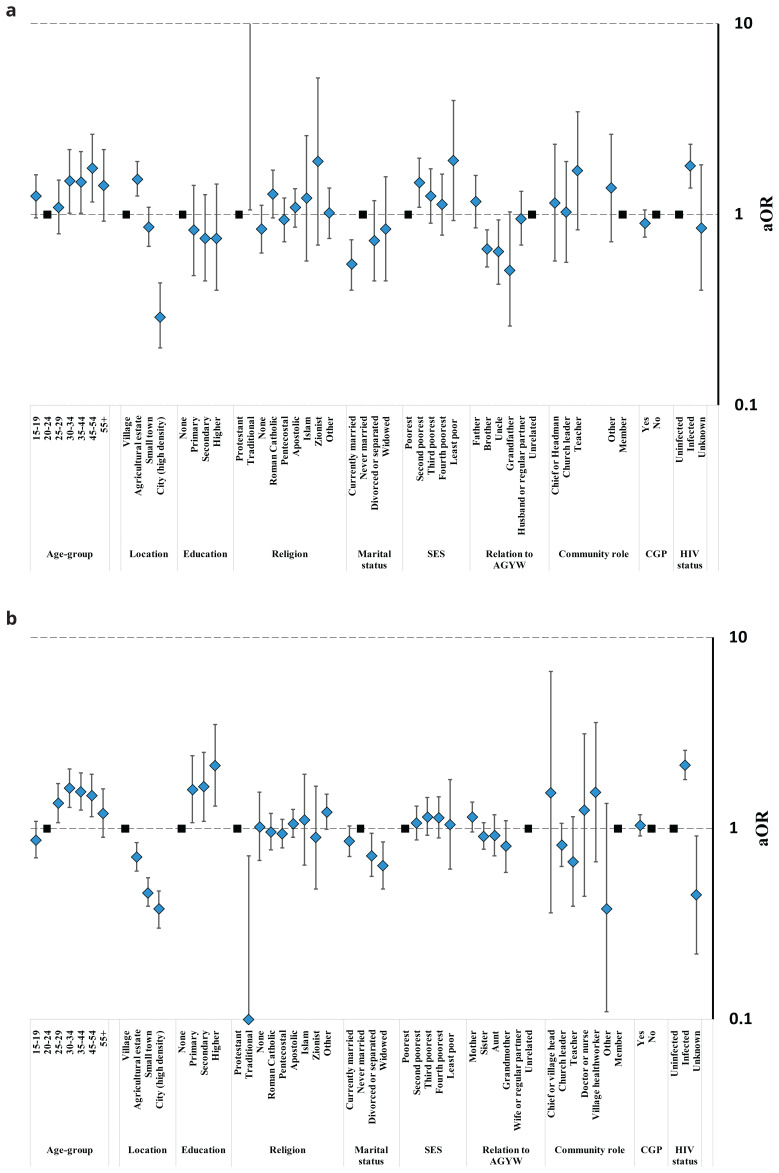
Fully-adjusted odds ratio (aOR; diamonds) and 95% confidence interval (whiskers) for acceptance of condom use in marriage by community location, relationship to an AGYW, and socio-demographic characteristics, Manicaland, Zimbabwe, 2018/2019. Graph ( **a**): Male community members. Graph ( **b**): Female community members.

Condom acceptability under all circumstances within marriage was lowest for men in city communities (6.7%), intermediate in small town (18.6%) and rural subsistence farming (21.7%) communities, and highest in agricultural estate communities (28.8%) ( Figure 6A); differences that were statistically significant after adjusting for other characteristics (Table S8). For women, there was a consistent urban-rural gradient in condom acceptability within marriage: lowest in city communities (20.7%), higher in small town communities (25.8%), higher again in agricultural estate communities (34.3%), and highest in rural subsistence farming communities (40.4%) ( Figure 6B, Table S8). Men and women aged 30–54 were more likely than younger people to support condom use in marriage. As with telling teenage daughters about condoms, for both men and women, those living with HIV were more likely to approve of condom use in marriage than uninfected people. After adjusting for other characteristics, men’s agreement with condom use in marriage varied with marital status (lowest agreement in currently married men) but not by education, socio-economic status, religion, community engagement or being in a community leadership role. Women’s agreement with condom use in marriage varied with marital status (highest agreement in never-married women and lowest agreement in divorced, separated and widowed women) and education (greater agreement at higher levels of education) but not by socio-economic status, religion, community engagement or having a community leadership role. Similar proportions of men with teenage daughters themselves and men not related to a teenage girl approved of condom use in marriage (26.5% *versus* 23.3%) but brothers (16.0%), uncles (17.6%) and grandfathers (13.6%) were all less likely to be in favour ( Figure 6A). More mothers (41.3%) than other women (31.3%) supported condom use within marriage but this difference was not statistically significant after also adjusting for community location and other individual-level characteristics (p=.13) ( Figure 6B). No evidence for differences was found for other female relatives of teenage girls.

### Influence of gender norms on condom use in AGYW at higher risk of HIV (AGYW ^@Risk^)

In this section, we look at how AGYW whose circumstances put them at risk of HIV infection (AGYW ^@Risk^) *perceive* other people's opinions (i.e. the community norms described above) to be and whether these perceptions influence their own views and use of condoms.

***HIV-negative AGYW ^@Risk^’s perceptions of the influence of gender norms and other structural factors on their decisions to use condoms by marital status.*** Similar proportions of unmarried and married HIV-negative AGYW ^@Risk^ reported perceiving negative community views (31.7% *versus* 29.2%), negative religious leaders views (27.4% *versus* 27.1%), and negative family views (27.7% *versus* 27.8%) that influenced their decisions on whether to use condoms with sexual partners ( Table 3). No differences were found in the proportions of HIV-negative AGYW ^@Risk^ reporting negative gender norms or family member views as influencing their decisions on condom use between those who were unmarried by single, divorced and widowed status or between those who were married by form of HIV risk but sample sizes were small (data not shown). Currently married HIV-negative AGYW ^@Risk^ were more likely than unmarried HIV-negative AGYW ^@Risk^ to believe that their peers were not using condoms (92.6% *versus* 69.3%).

**Table 3.** Proportions of unmarried and married AGYW at risk of HIV infection reporting perceptions or experience of unsupportive gender norms or structural barriers to their using male condoms.

Structural barrier	Unmarried AGYW		Married AGYW	
	Proportion (95% CI)	N	Proportion (95% CI)	N
**Community disapproval**	0.317 (0.253–0.386)	202	0.292 (0.247–0.340)	387
**Family disapproval**	0.277 (0.217–0.344)	202	0.278 (0.234–0.326)	385
**Religious leaders’ disapproval**	0.274 (0.213–0.341)	201	0.271 (0.227–0.318)	388
**Friends not using condoms**	0.693 (0.625–0.755)	205	0.926 (0.896–0.950)	394
**Pre-marital sex stigma**	0.385 (0.318–0.456)	205	-	-
**Condoms in marriage stigma**	-	-	0.736 (0.690–0.779)	394
**HIV stigma**	0.356 (0.290–0.427)	202	0.343 (0.296–0.392)	388
**Intimate partner violence**	0.166 (0.118–0.224)	205	0.218 (0.179–0.262)	394

**Legend:** 95% CI: 95% confidence interval.

 Perceived stigma around pre-marital sex (38.5%) and about use of condoms in marital relationships (73.6%) were reported by substantial proportions of HIV-negative unmarried and married AGYW ^@Risk^, respectively ( Table 3). Unmarried and married HIV-negative AGYW ^@Risk^ reported similar levels of HIV stigma (35.6% *versus* 34.3%) and intimate partner violence (16.6% *versus* 21.8%) ( Table 3). 

***Associations between gender norms and other structural barriers and AGYW ^@Risk^’s motivation, access and use of condoms.*** In the generic HIV prevention cascade explanatory framework, lack of social acceptability is hypothesised to be a barrier to use of HIV prevention methods by contributing to gaps in motivation ^31^. However, gender norms and associated structural factors could also contribute to gaps in access and capacity to use prevention methods. For example, where healthcare workers hold similar opinions on restricting young women’s sexual activity and access to prevention methods to those in the wider community or feel obliged to uphold these views.

**Figure 7.**
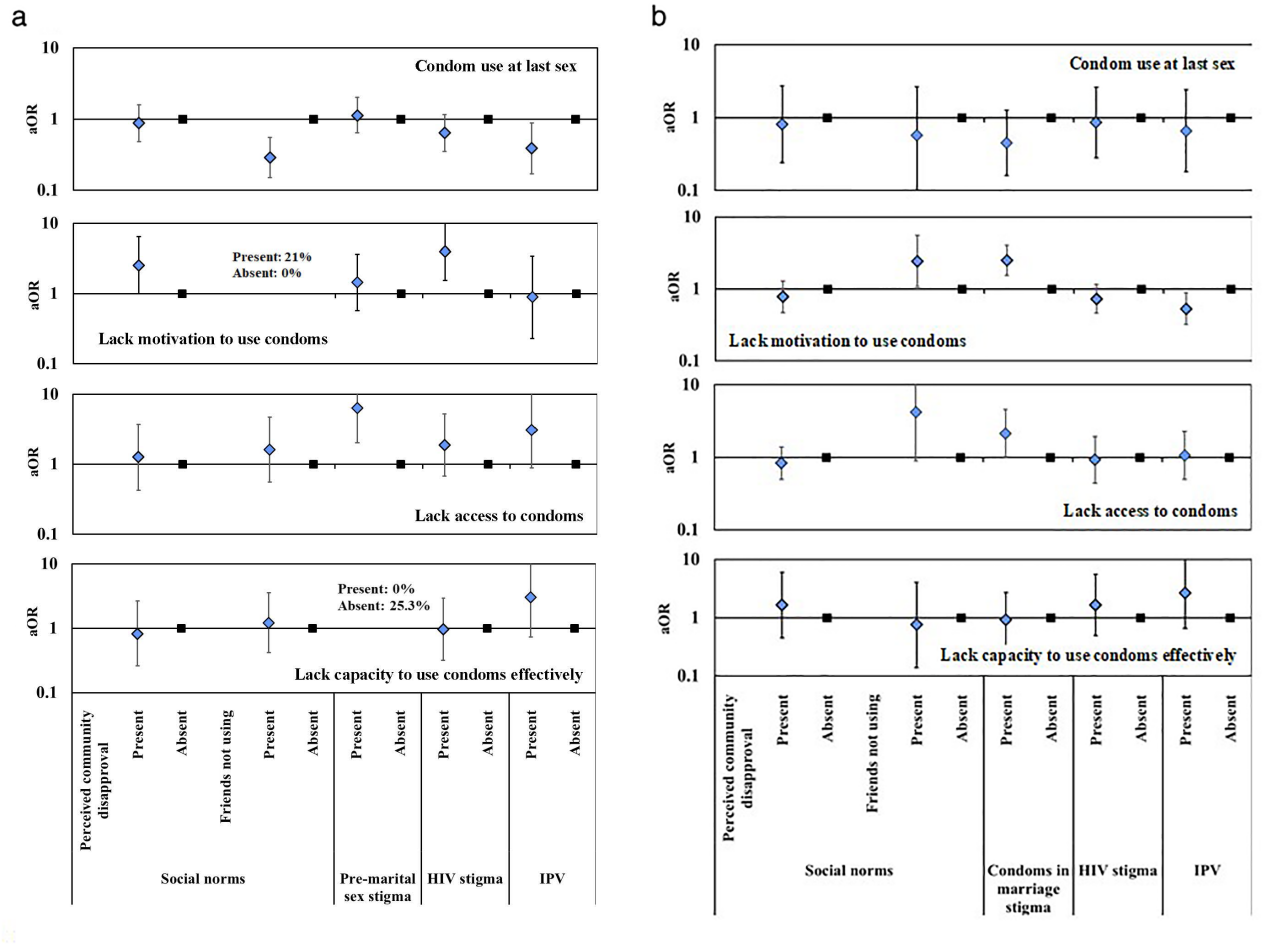
Age- and community location-adjusted odds ratios (aOR; diamonds) and 95% confidence intervals (whiskers) for condom use at last sex and for gaps in the condom HIV prevention cascade in AGYW at high risk of HIV infection reporting structural barriers to use of condoms, Manicaland, Zimbabwe, 2018/2019. Graph ( **a**): Unmarried AGYW at risk. Graph ( **b**): Married AGYW at risk.

In Figure 7, we show the results on associations between gender norms and potentially related structural barriers and gaps at each step in the HIV prevention cascades for condom use in unmarried and married AGYW ^@Risk^. 

For unmarried AGYW ^@Risk^, no association was found between perceived overall community disapproval of condom use and reported condom use at last sex (p=.69) ( Figure 7A). However, two-thirds (69.3%) of these AGYW reported that their friends were not using condoms and fewer of these women reported using condoms themselves than was the case for those who believed their friends were using condoms (39.4% *versus* 68.3%; p<.001). Looking closely at the gaps in the HIV prevention cascades for unmarried AGYW ^@Risk^, more of the young women who perceived community disapproval of their using condoms lacked motivation to use condoms (21.2%) than was the case for those who didn’t perceive community disapproval (10.3%) (p=.053); but no differences were found for lack of access (p=.68) or lack of capacity to use condoms effectively (p=.82). Twenty-one percent (22/105) of the AGYW in this group who thought their friends did not use condoms lacked motivation to use condoms themselves compared to none of those who said their friends did use condoms (N=56; Fishers exact test, p<.5). No differences were found between AGYW ^@Risk^ who did and did not think their friends were using condoms and lack of access (p=.38) or lack of capacity to use condoms effectively (p=.72). No differences were found according to whether unmarried AGYW ^@Risk^ felt there was pre-marital sex stigma (p=.67) or HIV stigma (p=.13) and condom use. However, reports of HIV stigma were positively associated with lack of motivation to use condoms in unmarried AGYW ^@Risk^ (24.1% *versus* 7.9%; p=.004); and, pre-marital sex stigma was positively associated with difficulties in accessing condoms in unmarried AGYW ^@Risk^ who wanted to use condoms (27.3% *versus* 6.0%; p=.001). Unmarried AGYW ^@Risk^ who experienced IPV were less likely to use condoms at last sex than those not experiencing IPV (29.4% *versus* 52.0%; p=.023). These women had similar levels of motivation to use condoms to their peers but tended to be more likely to report lack of access (26.3% *versus* 12.5% in motivated unmarried AGYW; p=.08) and lack of capacity to use (28.6% *versus* 15.2% in motivated unmarried AGYW with access; p=.13) condoms with their sexual partners. 

For married AGYW ^@Risk^, no associations were found between perceived overall community disapproval of condom use (p=.73) or believing friends were not using condoms (p=.48) and reported condom use at last sex ( Figure 7B). However, married AGYW ^@Risk^ who thought their friends did not use condoms were more likely to lack motivation to use condoms (58.4% *versus* 37.9%; p=.033) and, for those who were motivated, there was a trend towards more limited access (30.9% *versus* 11.1%; p=.07). Condom use in marriage stigma showed a weak association with non-use of condoms (p=.13) which appears to reflect greater lack of motivation to use condoms (63.1% *versus* 39.4%; p<.001) and, in motivated married AGYW ^@Risk^, greater lack of access (34.6% *versus* 19.0%; p=.048). No differences were found for married AGYW ^@Risk^ in condom use or in any of the gaps in the condom HIV prevention cascade between those who did and did not report community HIV stigma as a factor in their decisions to use condoms ( Figure 7B). A fifth of married AGYW ^@Risk^ reported recent IPV. These women were more likely than other married AGYW ^@Risk^ to be motivated to use condoms (55.8% *versus* 39.6%; p=.013); but, for those who were motivated and could access condoms, there was a trend towards lower capacity to use condoms effectively (8.8% *versus* 16.1%; p=.17).

## Discussion and conclusions

HIV incidence rates in AGYW in sub-Saharan Africa declined from the late 1990s to the mid-2010s but were still high at the end of this period ^8^ – raising severe life-long complications for the affected women and posing serious challenges to the sustainability of antiretroviral treatment programmes. Against this background, we described patterns of continuing support for traditional gender norms surrounding AGYW’s sexuality in high HIV burden communities in east Zimbabwe, and examined possible contributions of these norms to their risk of becoming infected with HIV using population survey data and an HIV prevention cascade framework. We found that 43.4% (79/182) of AGYW newly acquiring HIV infection between 1998 and 2013 were already married; 66% (394/599) of uninfected AGYW at higher risk of becoming infected between 2018 and 2019 were married; and male condom use was much lower in married AGYW ^@Risk^ than in unmarried AGYW ^@Risk^ (4.3% *versus* 61.5%). Much larger proportions of married AGYW ^@Risk^ than unmarried AGYW ^@Risk^ lacked motivation to use condoms and the capacity to use them effectively. Therefore, the study findings highlight *inter alia* the importance of recognising that AGYW ^@Risk^ are often already married and of distinguishing and addressing the differing circumstances and HIV prevention needs of these married and unmarried AGYW.

For unmarried AGYW ^@Risk^, those who thought their friends were not using condoms were less likely to use condoms themselves and – together with those who perceived community disapproval – were less likely to be motivated to use condoms. The overall gap in motivation was relatively small which suggests that gender norms may be less of a barrier to condom use in this group than we had anticipated. However, negative gender norms could also contribute to barriers to access or capacity to use condoms effectively. We did not find evidence for associations with friends not using condoms or perceived community disapproval – possibly due to small sample sizes – but many unmarried AGYW ^@Risk^ reported pre-marital sex stigma as a barrier to their being able to access condoms.

The bigger gap in motivation for married AGYW ^@Risk^ than for unmarried AGYW ^@Risk^ reflected low social acceptability of condom use within marriage but also married AGYW ^@Risk^’s lower levels of personal risk perception, poorer knowledge about condoms, and greater concerns about the negative consequences ^2^. Low personal risk perception in AGYW ^@Risk^ has been noted in other studies in sub-Saharan Africa ^38^ and, in Manicaland, longitudinal associations have been found between changes in HIV risk perception and changes in condom use ^39^.

The gaps in the HPCs between motivation and access to condoms were quite small for both unmarried and married AGYW ^@Risk^ with concerns about the acceptability of provision – reflecting worries about privacy and confidentiality and being embarrassed to request condoms – being the main reason for these gaps in both groups.

### Waning influence of traditional gender norms on unmarried AGYW’s sexuality and condom use?

Almost a quarter (24.4%) of unmarried AGYW interviewed in this study reported having started sex with the great majority of those currently sexually active and at risk of HIV infection being motivated to use condoms. We found a substantial degree of community acceptance of this pattern of behaviour: that most young women had sex before marriage nowadays and, to a lesser but still considerable extent, that this was acceptable and that teenage girls should be told about condoms and given access to them in schools. These results indicate that the traditional gender norm against female pre-marital sex has probably been weakening in Manicaland; a finding broadly in line with findings from studies in other parts of sub-Saharan Africa ^20, 40^. Nevertheless, those AGYW ^@Risk^ who reported community disapproval (31.7%) or friends not using condoms (69.3%) were less likely to be motivated to use condoms than their peers which suggests that traditional social norms and peer pressure remain important influences on many unmarried AGYW’s sexual activity and condom use. Interestingly, the widespread perception amongst unmarried AGYW ^@RISK^ that most young women in their situation are not using condoms appears to be a misconception as 61.5% of these women reported that they *did* use condoms when they last had sex. Therefore, this barrier to wider condom use could be rectified in interventions. HIV stigma, experienced or perceived by unmarried AGYW ^@Risk^, was also associated with their lacking motivation to use condoms ^41^ and may be reinforcing these effects. A sizeable minority (16.6%) of unmarried AGYW ^@Risk^ reported having experienced IPV which – as was the case in an earlier study in Johannesburg and Harare ^42^ – was associated with less condom use. In Manicaland, we also found that this was linked to greater difficulties accessing condoms and to lower capacity to use them effectively.

Some community members’ or groups’ views may have particularly strong or more limited influence on AGYW’s sexual behaviour and use of condoms. Generally, we found that female community members were less likely than their male counterparts to find it acceptable for their daughters to have sex before marriage and to have access to condoms. Women who were mothers of teenage daughters at the time of the survey had similar views to women as a whole; however, aunts of teenage girls, who – in the *Shona* culture which predominates in Manicaland – play an important role in helping young women to navigate marital arrangements, expressed more liberal views on pre-marital sex. Fathers, and male relatives generally, were more likely than other men to be against female pre-marital sex. In in-depth interviews and focus group discussions, held in the same study areas in Manicaland, parents reported conflicting attitudes towards pre-exposure prophylaxis (PrEP)’s being made available for AGYW ^43^. We found that while ‘… parents wanted to see girl-children protected from HIV … they struggle to reconcile this with traditional ‘good girl’ notions that stigmatise pre-marital sex’. As a consequence, parents often ‘co-produce public gender norms that prevent AGYW from accessing PrEP’ and ‘AGYW require permission and experience limited autonomy to use PrEP due to intersecting sexuality- and PrEP-related stigmas’ ^44^. It seems likely that parents experience similar mixed feelings towards their daughters having access to condoms.

AGYW ^@Risk^ – in common with most other members of their communities – reported that ‘many young women have sex before marriage these days’. In these circumstances, young women might be expected to experience strong peer pressure to become sexually active especially given the opportunities to obtain material benefits that early sexual activity can bring ^10, 45, 46^. However, younger and older AGYW ^@Risk^ differed in their views on this: whilst relatively few teenage AGYW ^@Risk^ supported pre-marital sex and AGYW’s having access to condoms, those aged 20–24 had similar views on pre-marital sex to older women and were more likely to approve of their having access to condoms. Therefore peer pressure to engage in pre-marital sex in Manicaland may become stronger as young women get older. Two-thirds of unmarried AGYW ^@Risk^ said that friends and community members’ views either encouraged them to use condoms or were unimportant. In a systematic review of studies investigating peers influence on young people’s sexual behaviour in sub-Saharan Africa, Fearon and colleagues identified 11 studies that tested 37 possible associations ^47^. They found evidence for an association between peers and sexual behaviour for 51% (19/37) of the associations tested but noted that longitudinal designs with biomarker outcomes were needed to provide conclusive evidence on the role of peers in adolescent behaviour.

A quarter (27.4%) of unmarried AGYW ^@Risk^ said that church leaders had a strong influence on their use of condoms. More male church leaders than other men in the community were against the idea of young women having pre-marital sex but they didn’t differ in their levels of support for making condoms available for young women. Female church leaders were actually more in favour of this than other women. Church leaders views on young women’s early sexual activity could vary between religious denominations. Sample sizes were too small for this to be investigated directly using the survey data but there was no evidence for differences in the population as a whole: members of all churches were less likely to support female pre-marital sex than people with no religion. Members of Apostolic churches were less likely to support making condoms available to unmarried young women than people subscribing to other religions ^48^.

Many young women in Manicaland are members of self-assessed ‘well-functioning’ local community groups ^49^. In previous studies, we have seen that participation in these groups can contribute to social changes (e.g. early adoption of safer sexual behaviours ^50^ and uptake of new HIV services including HIV testing and prevention of mother-to-child transmission ^51^). In the current study, we found that women who were members of community groups were more likely to accept unmarried AGYW starting sex and having access to condoms. Therefore community group participation may be one pathway through which traditional social norms on young women’s sexual activity are being eroded.

The views of local healthcare workers may also be an important influence on unmarried AGYW ^@Risk^‘s access to HIV prevention methods. If they disapprove of female pre-marital sex, this will probably contribute to AGYW ^@Risk^s’ sense that they can’t access condoms due to lack of acceptable provision. In the survey, nurses and community health workers (who are both largely female) were more likely to express supportive views towards AGYW having access to condoms than other women in the community. However, in in-depth interviews, some were self-critical of how they, as a group, interacted with AGYW. Those in rural areas, especially, acknowledged how their community-embeddedness affected their perceptions of AGYWs’ sexuality ^34^. This mix of community-embeddedness and self-awareness may help to explain the patterns we found in the condom cascade for unmarried AGYW ^@Risk^ – i.e. a relatively small gap between the motivation and access bars in the cascade but lack of acceptable provision being the largest (>75%) barrier contributing to this gap.

### Resilient gender norms on married AGYW’s condom use?

Only a small minority of married AGYW ^@Risk^ (4.3%; 17/394) reported using condoms when they last had sex. Six of these women reported using condoms as a family planning method and two had multiple sexual partners themselves and may have had their last sex with a non-regular partner. Therefore, underlying levels of condom use for HIV prevention within marriage are probably even lower than 4%. If so, this would be consistent with the limited community support we found for condom use generally within marriage (34.1%), the high condom in marriage stigma reported by married AGYW ^@Risk^ (73.6%), and the small minority of these young women who think their friends use condoms (7.4%). Reports of condom in marriage stigma and believing that friends are not using condoms were both associated with less motivation to use condoms themselves ^3^ which could be indicative of peer pressure. However, only a minority (29.2%) of married AGYW ^@Risk^ felt that community disapproval was an obstacle to their using condoms and no difference was found in motivation to use condoms between those who did and did not report community disapproval as being an obstacle. Therefore, other factors, such as low risk perception and perceived negative consequences of using condoms (e.g. loss of sexual pleasure and fertility desires) may be stronger barriers to these women’s being motivated to use condoms.

Community views on condom use within marriage were less favourable in cities than in rural areas; perhaps surprisingly as rural communities might have been expected to be more conservative and HIV prevalence, typically, has reached higher levels in urban and peri-urban areas ^52, 53^. Within the study communities, as elsewhere in sub-Saharan Africa, younger people (15–24 years) were less likely than older people (≥25 years) ^40^ and currently married people were less likely than never-married people to support condom use in marriage ^54^. This may be, in part, because this is a demographic who are keen to have children or feel that their risk of HIV infection is low. As a consequence, any influence from married AGYW ^@Risks^ peer groups would tend to affirm and reinforce their adversity to condom use. Parents of teenage daughters and other potentially influential community members expressed similar views to the wider communities in which they lived.

Married AGYW ^@Risk^ who reported having experienced IPV were equally likely to report condom use as those who did not report IPV. Whilst more of these women wanted to use condoms, those who were motivated to do so showed a trend towards lower capacity to use condoms. In an earlier analysis of a wider age-range (ages 15–54) of married women in Manicaland, we found a positive association between experiencing IPV and being at risk of HIV infection (72.3% *versus* 58.5%; p<.001); but, as here with married AGYW ^@Risk^, no difference in condom use amongst those at risk ^55^. The HIV prevention cascades for women reporting and not reporting IPV were similar; both showing large gaps in motivation and capacity to use condoms. Women reporting motivation and access were more likely to report their partner being a barrier to condom use if they experienced IPV (84.8% *versus* 75.5%; p=.015).

### Strengths and limitations

Comins and colleagues conducted a survey of AGYW recruited from selected venues using time-location sampling and used latent class analysis to identify intersecting social- and structural-level determinants of HIV/STI acquisition in Ethiopia ^56^. Previous qualitative studies have described community views on aspects of AGYW’s sexual behaviour ^4^ with some finding evidence for changing community views on AGYW sexuality and use of HIV prevention methods ^20^. However, to the best of our knowledge, this is the first study to measure contemporary support for traditional gender norms surrounding young women’s sexuality in a representative population survey, in a high HIV prevalence sub-Saharan Africa setting, and to investigate their influence on condom use for HIV prevention.

The study had a good sample size with a good participation rate, was conducted in a mix of different socio-economic settings (making the findings generalisable to much of Zimbabwe), used biomarker data to identify uninfected individuals, and utilised bespoke validated HPC data collected in the survey that covered multi-level demand and supply side barriers to condom use ^31^. The data analysis was stratified by marital status which highlighted important differences in the gaps and barriers to condom use between these two groups of AGYW at higher risk of HIV infection. The survey data used in the main analyses for the study were cross-sectional so we have not been able to establish the direction or causality of the associations we observed. Longitudinal studies are needed to quantify changes in support for and adherence to traditional and emerging new social norms over time. Furthermore, the population cohort survey data used to compare rates of new infections in unmarried *versus* married AGYW were only available for an earlier period (1998–2013) so we can’t be sure that the observed relative levels of HIV incidence were still valid in 2018/19. Informal confidential voting interview methods ^57^, adapted for use with questionnaires administered using tablets in the 2018/19 survey, were used to reduce social desirability bias in the data on sexual behaviour. However, some residual under-estimation of the priority populations for HIV prevention methods is likely and the measurements of social norms and other structural barriers also will be subject to this bias. When populating the HPCs, we assumed that the AGYW who reported using condoms were doing so – at least in part – for HIV prevention and were motivated and had access to condoms for this purpose. Where AGYW ^@Risk^ reported using condoms for family planning, this assumption may not have been correct; in which case, our estimates for all bars in the HPCs will have been overstated.

PrEP is an additional HIV prevention option available now for AGYW ^@Risk^ in the general population in Zimbabwe and elsewhere in sub-Saharan Africa ^58^. The current study was limited to condoms; but, despite PrEP’s fundamental advantage in being a method over which women can, in principle, exercise direct control, there are early signs that the norms that we have examined here can also act as obstacles to AGYW’s use of PrEP ^44, 59^. 

### Implications for HIV prevention in sub-Saharan Africa

In an analysis of Zimbabwe Demographic and Health Survey data, Sambisa and colleagues ^60^ described differences in AGYW’s sexual risk behaviours for HIV infection (SRBs) between the Shona and Ndebele tribes and concluded that condom promotion approaches could be strengthened by recognising and responding to cultural forces that reproduce and perpetuate risky sexual behaviours. In a community-randomised control trial in South Africa, Pettifor and colleagues found that a community mobilization intervention reduced negative gender norms in men but did not reduce IPV or increase condom use ^3^. The authors concluded that more time might be needed to change behaviour or that interventions may need to address behaviours more directly. Following an analysis of survey data collected from sexually-experienced students in Nyanza, Kenya – and consistent with our HIV prevention cascade framework – Maticka-Tinsdale and Eric Tenkorang ^17^ concluded that key factors influencing reported condom use existed at the school/community levels as well as at the individual level. They recommended that HIV prevention interventions should take account of barriers at each of these levels.

Based on the findings from these and other studies, the PEPFAR-supported multi-country DREAMS programme, developed to reduce SRB and increase AGYW’s use of HIV prevention methods, incorporated activities to address barriers at community, sexual partner and individual levels ^61^. Community-strengthening programmes included community conversations with male opinion leaders to promote positive gender norms and IPV reduction activities. Early findings from the comprehensive set of planned DREAMS evaluations show mixed evidence for an impact of the programme in reducing new infections – with faster reductions in new diagnoses in pregnant women in settings where more DREAMS activities have been implemented ^62^ but no evidence for an acceleration in HIV incidence declines in the period immediately following their introduction ^7^. This may be because early uptake of community activities was low in some intervention settings ^63^. However, whilst our findings support the overall layered approach adopted in the DREAMS programme, they suggest a need to establish social spaces where helpful norms can be cemented, unhelpful norms (e.g. moralistic attitudes and conservative gender ideologies) renegotiated, and popular misconceptions (e.g. on levels of condom use amongst unmarried AGYW ^@Risk^) challenged. This can be achieved through community conversations, an approach that provides community members with a platform to come together to share perspectives and collectively address community challenges (like those discussed above) ^64, 65^. In other health contexts, where gender inequalities have constituted a problem, community conversations have proved productive in transforming gender norms and practices ^66^.

## Data Availability

Due to the sensitive nature of data collected, including information on HIV status, treatment and sexual risk behaviour, the Manicaland Centre for Public Health does not make full analysis datasets publicly available. Summary datasets of household and background sociodemographic individual questionnaire data, covering rounds 1–8 (1998–2021), are publicly available for download via Manicaland Centre for Public Health. Additionally, summary HIV incidence and mortality data spanning rounds 1–6 (1998–2013), created in collaboration with the ALPHA Network are available via the DataFirst Repository. Further quantitative data used for analyses produced by the Manicaland Centre for Public Health are available on request following completion of a data access request form. Zenodo: Gregson_et_al_HIV_young_women_social_norms_2024_extended_data https://doi.org/10.5281/zenodo.10514520 ^67^ This project contains the following extended data: Gregson_et_al_HIV_young_women_social_norms_2024_Extended_data.pdf (Detailed data and survey questions) Data are available under the terms of the Creative Commons Attribution 4.0 International license (CC-BY 4.0).

## Footnotes

^1^ We did find a statistically significant association for men in agricultural estates being more likely than those in rural communities to agree that providing condoms in schools was acceptable (58.5% *versus* 48.2%; p=.003). In general, the response patterns for this latter question were broadly similar to those on telling teenagers about condoms; exceptions including that both fathers and mothers were more in favour than their unrelated counterparts and there were no longer any differences by community engagement.

^2^ In this analysis, the negative consequences variable was limited to AGYW’s concerns that use of condoms could affect their own sexual pleasure. Other perceived negative consequences of condom use could include their contraceptive effects for AGYW wishing to have children.

^3^ Condom in marriage stigma and believing that friends are not using condoms were also both weakly associated with barriers to access to condoms ( Figure 6B).

^4^ For example, Cockcroft and colleagues on inter-generational sexual relationships in urban and rural areas in Botswana, Namibia and Swaziland 45. Cockcroft A, Kunda JL, Kgakole L, Masisi M, Laetsang D, Ho-Foster A, *et al.* **Community views on inter-generational sex: findings from focus groups in Botswana, Namibia and Swaziland**. *Psychology, Health and Medicine* 2010; 15(5):507–514.; and Wamoyi and colleagues on transactional sex in Tanzania 46. Wamoyi J, Helse L, Melksin R, Kyegombe N, Nyato D, Buller AM. **Is transactional sex exploitative? A social norms perspective, with implications for interventions with adolescent girls and young women in Tanzania**. *Public Library of Science One* 2019; 14(4):e0214366.
