# Supplementary material for: Gender Norms and Structural Barriers to Use of HIV Prevention in Unmarried and Married Young Women in Manicaland, Zimbabwe: An HIV Prevention Cascade Analysis
Source: Gates Open Res. 2025 May 27;8:22. Originally published 2024 Mar 7. [Version 2] doi: 10.12688/gatesopenres.15127.2 (PMC12397500; doi:10.12688/gatesopenres.15127.2)
Supplement: Peer review history

# Reviewer report: Reviewer response for version 2

**DOI:** 10.21956/gatesopenres.17714.r39670

**Published:** 2025-08-29

**Authors:** Ashley L. Buchanan, Claire Pearsall

**Affiliations:** 1 Department of Pharmacy Practice and Clinical Research, University of Rhode Island College of Pharmacy, Kingston, Rhode Island, USA; 2 University of Rhode Island College of Pharmacy, Kingston, Rhode Island, USA

Review of “Gender Norms and Structural Barriers to Use of HIV Prevention in Unmarried and Married Young Women in Manicaland, Zimbabwe: An HIV Prevention Cascade Analysis”

 General Comments

 The authors present the findings from an HIV prevention cascade analysis on gender norms and structural barriers to use of HIV prevention in unmarried and married young women in Manicaland, Zimbabwe. This article was overall well-written, and an interesting contribution to the literature on this topic. We had one major comment about the survey sampling design and methods. Please find major and minor comments below.

 Specific Comments for Revision

 Major Comments

 Were survey weights used to ensure inference in the underlying population? If so, those need to be described. If not, why not? Were the survey questions to measure support for condom use in marriage validated, was it developed in previous literature, or is this the first time it’s being used?

 Minor Comments

            

1. Abstract:
  1. Make sure to clearly define HIV prevention cascade.
  2. The conclusion of the abstract states, “Negative gender norms can form a barrier,” which may be perceived as causal language, given that the authors conducted a cross-sectional study that cannot establish causality or the direction of the associations.
2. Introduction:
  1. Would it need to be a serodiscordant partnership for HIV transmission risk (rather than among HIV-negative partners)?
  2. What about transactional sex as a risk factor for HIV transmission?
  3. Can HIV testing and treatment be considered a prevention strategy, because it prevents onward transmission?
  4. Make sure to define HIV prevention care cascade somewhere in the paper.
  5. Overall, the introduction is clear and motivates the study, and we also like the clearly defined objectives of the study.
3. Methods:
  1. Why were only 2/3 of those who spent the night in the households before the census visits invited to participate?
  2. What was the response rate to the survey?
  3. Should a sensitivity analysis be done for the date of seroconversion? Taking the last known negative date and first known positive date?
4. Results:
  1. The results are interpreted odds, but no odds ratios are reported. Add odds ratios and 95% confidence intervals.
  2. In the section about social norms for married AGYW, it may be worth reminding the reader where the model results begin and what was adjusted for in the model.
  3. Report the odds ratios and 95% confidence intervals in the section about condom acceptability.
  4. When you say “Influence of gender norms on condom use in AGYW at higher risk of HIV,” influence sounds like causal language. How is influence defined? Please clarify this is an association.
  5. Please report point estimates and 95% confidence intervals rather than just p-values.
  6. Perhaps you should move some of the results to an appendix, as it was a bit lengthy.
5. Discussion:
  1. It could be interesting to look at spillover of condom use between social contacts in this population in the future.
  2. How is influence defined? It implies a causal influence.
  3. You may not need to rehash the results in the discussion as much, since they are already reported in the results section.
  4. In the strengths and limitations section, are there any differences in this setting and Ethiopia to be aware of? What survey sampling methods were used in the Ethiopia study to ensure it was generalizable?
  5. In addition to allowing more time for behavior change interventions and addressing behaviors more directly, one could also potentially do a network intervention with a peer education model. Network studies could be interesting in the future.
6. Tables and Figures:
  1. Table 1: Is the table showing the proportion of people who agreed or disagreed with the statements? You could also move N to the top of the table.
  2. Figure 1: The figure looks a little bit distorted. Should the authors test for the trend on figure A?
  3. Figure 3a: Some of these groups are small, worth noting as a limitation.
  4. Figure 4: Some of your confidence limits are off the chart, what are the minimum and maximum?
  5. Overall we like the plots in figure 4a and 4b, because they make it easy to view a lot of results quickly.
  6. Figure 7: You may not need the reference group in the figure.
7. Other:
  1. What is this link? https://doi.org/10.5281/zenodo.1051452065
  2. What does this comment from Lucy Chimoyi refer to? “The explanation for hyperinflation is not necessary in the results section. Ensure that the tables and graphs are self-explanatory.”

Is the work clearly and accurately presented and does it cite the current literature?

Yes

If applicable, is the statistical analysis and its interpretation appropriate?

Partly

Are all the source data underlying the results available to ensure full reproducibility?

Yes

Is the study design appropriate and is the work technically sound?

Partly

Are the conclusions drawn adequately supported by the results?

Yes

Are sufficient details of methods and analysis provided to allow replication by others?

Partly

Reviewer Expertise:

Biostatistics, Causal Inference, Generalizability, Interference, HIV prevention, substance use

We confirm that we have read this submission and believe that we have an appropriate level of expertise to confirm that it is of an acceptable scientific standard, however we have significant reservations, as outlined above.

# Reviewer report: Reviewer response for version 1

**DOI:** 10.21956/gatesopenres.16463.r36677

**Published:** 2024-07-23

**Authors:** Maxime Inghels

**Affiliations:** 1 University of Lincoln, Lincoln, UK

This is a very interesting article that aims to investigate gender norms related to HIV prevention, specifically focusing on condom use. While this topic has been previously studied using qualitative methods, this article is one of the few, if not the first, to investigate this aspect using a quantitative approach, making it a valuable contribution to the field. The author shows that social norms are one of the main barriers to condom use motivation among AGYW populations. They also demonstrate a link between the perception of social norms and condom use among unmarried AGYW populations.

 Although the results section is a bit lengthy, I found the article to be clear and well-written. I have a few minor comments and suggestions that might help improve the clarity of the manuscript.

**Abstract**

 The method mentions a logistic regression model to measure associations between AGYW’s perception of negative gender norms and HIV prevention cascades involving condom use, but the related results are not presented in the results section of the abstract.

 Since the results presented can refer to different populations (e.g., men, women, AGYW, unmarried AGYW at risk, married AGYW at risk), the sample size of each population should be presented or at least the ratio with numbers associated with each percentage should be added.

**Introduction**

 The explanation of the context is clear and well-written. My only comment is about the summary of the findings at the end of the section, which is a bit unusual to me.

**Methods**

 The authors have clearly presented what they have done. I did not find any missing elements that could prevent the understanding of their methodology.

**Results**

 “Eighty-six point three percent (95% CI, 80.1%–81.2%) of unmarried AGYW at risk” – the percentage does not fall within the confidence interval.

 As the authors consider multiple populations, which can sometimes be hard to follow, I would encourage them to add the headcount fraction associated with each percentage presented in the text.

 Sub-section title p12: “HIV-negative AGYW@Risk’s perceptions of the influence of gender norms and other structural factors on their decisions to use condoms by marital status.” I would drop the term “HIV-negative” as it is misleading since all the AGYW populations considered in the study are HIV-negative.

**Discussion**

 I have no comments on this section.

**Discretionary General Comments**

 I would encourage the author to refer to the UNAIDS terminology guidelines, particularly for the use of “exposed” instead of “at risk” population (see: UNAIDS Terminology Guidelines).

Is the work clearly and accurately presented and does it cite the current literature?

Yes

If applicable, is the statistical analysis and its interpretation appropriate?

Yes

Are all the source data underlying the results available to ensure full reproducibility?

Yes

Is the study design appropriate and is the work technically sound?

Yes

Are the conclusions drawn adequately supported by the results?

Yes

Are sufficient details of methods and analysis provided to allow replication by others?

Yes

Reviewer Expertise:

HIV, Epidemiology, Demography.

I confirm that I have read this submission and believe that I have an appropriate level of expertise to confirm that it is of an acceptable scientific standard.

# Response

**Published:** 2025-04-06

**Authors:** Simon Gregson

**Affiliations:** School of Public Health, Imperial College London, London, England, UK

We thank the reviewer for the very helpful comments and suggestions for improvements to the manuscript. We have updated the manuscript now in the ways described in the responses to each comment listed below: 

Abstract

 The method mentions a logistic regression model to measure associations between AGYW’s perception of negative gender norms and HIV prevention cascades involving condom use, but the related results are not presented in the results section of the abstract.

  

- Response: The results section of the Abstract includes results from these logistic regression analyses. For example, the p-values given for the comparisons of condom use according to whether or not friends use condoms are results from these analyses. The journal’s abstract word limit restricts the detail we can provide here; but, to make this more apparent, we have edited the text and replaced the p-values with odds ratios (with 95% confidence intervals). There wasn’t enough space in the Abstract to include results from the logistic regression analyses investigating socio-demographic variations in community views on AGYW’s use of condoms so we have removed mention of this from the Methods section.

Since the results presented can refer to different populations (e.g., men, women, AGYW, unmarried AGYW at risk, married AGYW at risk), the sample size of each population should be presented or at least the ratio with numbers associated with each percentage should be added.

  

- Response: We agree with the reviewer that including more information on sample sizes for the different parts of the analysis would be helpful. Unfortunately, this isn’t feasible given the journal’s 300 word limit for the abstract.

Introduction

 The explanation of the context is clear and well-written. My only comment is about the summary of the findings at the end of the section, which is a bit unusual to me.

  

- Response: Please see our response to the similar comment by Reviewer 1.

Results:

 “Eighty-six point three percent (95% CI, 80.1%–81.2%) of unmarried AGYW at risk” – the percentage does not fall within the confidence interval.

  

- Response: The upper bound of the confidence interval should have been ‘91.2%’ and has been corrected in the text.

As the authors consider multiple populations, which can sometimes be hard to follow, I would encourage them to add the headcount fraction associated with each percentage presented in the text.

  

- Response: In most cases, the numerators and denominators are shown in the Tables in ‘Extended data’ but we agree with the reviewer that it would be easier for the readers if they are shown in the text and so we have done this now. In the remaining cases, these details are provided in Tables in the paper; so, to avoid over-cluttering the text, we have not repeated them there.  

Sub-section title p12: “HIV-negative AGYW@Risk’s perceptions of the influence of gender norms and other structural factors on their decisions to use condoms by marital status.” I would drop the term “HIV-negative” as it is misleading since all the AGYW populations considered in the study are HIV-negative.

  

- Response: ‘HIV-negative’ has been dropped now from this sub-section title.

Discretionary General Comments:

 I would encourage the author to refer to the UNAIDS terminology guidelines, particularly for the use of “exposed” instead of “at risk” population (see: UNAIDS Terminology Guidelines).

  

- Response: We have referred to the UNAIDS terminology guidelines. However, given the focus of this particular scientific paper, we believe it is important to be as precise as possible about the meanings of the variables that are being used. Therefore we prefer to retain the current use of ‘at risk’ populations.

# Reviewer report: Reviewer response for version 1

**DOI:** 10.21956/gatesopenres.16463.r36328

**Published:** 2024-07-19

**Authors:** Lucy Chimoyi

**Affiliations:** 1 Implementation Research, The Aurum Institute, Johannesburg, Gauteng, South Africa

This topic is very relevant in such settings where traditional gender norms influence adoption of HIV prevention practices among young women, whether married or unmarried. The authors have gone to great lengths to show where the gaps exist and provided a solution in addressing the gap. I have provided a point-by-point review of the article below:

 Introduction

 The last paragraph of the introduction is providing methods, results, and some interpretation of some of these findings. Something I find unusual. Usually, the last paragraph focusses on the gap and what was done by the authors to address this important gap. My suggestion is to move some of the information in this paragraph to the appropriate sections.

 Methods

 Study settings: why were these particular 8 communities selected?

 The paragraph beginning with “Following these procedures….” Is reporting some findings which should be moved to the Results Section.

 In the methods, authors mention the survey period as between July 2018 and December 2019 and also introduced data between 1998-2013 which included 6/8 communities. Authors have not explained how they established the calculated risk difference for the two communities not included in the previous survey. Did they consider estimating the parameters using available data from published peer-review articles or grey literature? Or is this a limitation?

 Results

 The explanation for hyperinflation is not necessary in the results section.

 Ensure that the tables and graphs are self-explanatory. Write in full (footnotes) all acronyms as some are not universally known such as CGP.

 Introduction

 The last paragraph of the introduction is providing methods, results, and some interpretation of some of these findings. Something I find unusual. Usually, the last paragraph focusses on the gap and what was done by the authors to address this important gap. My suggestion is to move some of the information in this paragraph to the appropriate sections.

 Methods

 Study settings: why were these particular 8 communities selected?

 The paragraph beginning with “Following these procedures….” Is reporting some findings which should be moved to the Results Section.

 In the methods, authors mention the survey period as between July 2018 and December 2019 and also introduced data between 1998-2013 which included 6/8 communities. Authors have not explained how they established the calculated risk difference for the two communities not included in the previous survey. Did they consider estimating the parameters using available data from published peer-review articles or grey literature? Or is this a limitation?

 Results

 The explanation for hyperinflation is not necessary in the results section.

 Ensure that the tables and graphs are self-explanatory. Write in full (footnotes) all acronyms as some are not universally known such as CGP.

 Discussion

 The first paragraph summarized the findings of the paper without providing a background interpretation. This interpretation can be done in the subsequent paragraphs in the discussion.

 Can the authors clarify the experienced stigma in their discussion? Reference to this form of stigma is missing from the results. This was mentioned on page 16 from this sentence " *HIV stigma, experienced or perceived by unmarried AGYW@Risk, was also associated with their lacking motivation to use condoms41 and may be reinforcing these effects.”*

Is the work clearly and accurately presented and does it cite the current literature?

Yes

If applicable, is the statistical analysis and its interpretation appropriate?

Yes

Are all the source data underlying the results available to ensure full reproducibility?

Yes

Is the study design appropriate and is the work technically sound?

Yes

Are the conclusions drawn adequately supported by the results?

Yes

Are sufficient details of methods and analysis provided to allow replication by others?

Yes

Reviewer Expertise:

Infectious disease epidemiology, Implementation science, evaluation and qualitative research

I confirm that I have read this submission and believe that I have an appropriate level of expertise to confirm that it is of an acceptable scientific standard, however I have significant reservations, as outlined above.

# Response

**Published:** 2025-04-06

**Authors:** Simon Gregson

**Affiliations:** School of Public Health, Imperial College London, London, England, UK

We thank the reviewer for the very helpful comments and suggestions for improvements to the manuscript. We have updated the manuscript now in the ways described in the responses to each comment listed below: 

Introduction:

 The last paragraph of the introduction is providing methods, results, and some interpretation of some of these findings. Something I find unusual. Usually, the last paragraph focusses on the gap and what was done by the authors to address this important gap. My suggestion is to move some of the information in this paragraph to the appropriate sections.

  

- Response: This approach is often used in social science articles similar in length and nature to the current paper. However, as both reviewers have suggested we change it here, we have replaced the last paragraph of the Introduction with a shorter list of objectives.

Methods:

 Study settings: why were these particular 8 communities selected?

  

- Response: The 8 communities were selected to reflect the main socio-economic population strata found in Zimbabwe. We have clarified this now in the text in the ‘Study setting and survey procedures’ sub-section of the Methods section.

The paragraph beginning with “Following these procedures….” Is reporting some findings which should be moved to the Results Section.

  

- Response: These details have been moved now to the Results section.

In the methods, authors mention the survey period as between July 2018 and December 2019 and also introduced data between 1998-2013 which included 6/8 communities. Authors have not explained how they established the calculated risk difference for the two communities not included in the previous survey. Did they consider estimating the parameters using available data from published peer-review articles or grey literature? Or is this a limitation?

  

- Response: The risk difference between the communities included in the current and previous surveys could not be calculated because no representative general population data on HIV incidence exist for the two new communities. We have noted this limitation now in the Discussion.    

Results:

 The explanation for hyperinflation is not necessary in the results section.

  

- Response: The reference to hyperinflation has been removed from the Results section. 

Ensure that the tables and graphs are self-explanatory. Write in full (footnotes) all acronyms as some are not universally known such as CGP.

  

- Response: We have reviewed the footnotes for all tables and graphs in the paper and in the extended data and have elaborated the acronyms to make them self-explanatory.

Discussion:

 The first paragraph summarized the findings of the paper without providing a background interpretation. This interpretation can be done in the subsequent paragraphs in the discussion.

 • Response: We agree with the reviewer and this is indeed the approach that we have taken in the             Discussion.

 Can the authors clarify the experienced stigma in their discussion? Reference to this form of stigma is missing from the results. This was mentioned on page 16 from this sentence " *HIV stigma, experienced or perceived by unmarried AGYW@Risk, was also associated with their lacking motivation to use condoms41 and may be reinforcing these effects.”*

  

- Response: The reviewer is quite correct, the results in this paper are just for perceived stigma. We have removed the mention of experienced stigma now from the text on page 16.
